# Analysing the effectiveness of topical bleeding care following tooth extraction in patients receiving dual antiplatelet therapy-retrospective observational study

**DOI:** 10.1186/s12903-021-01391-9

**Published:** 2021-01-15

**Authors:** Bogumił Lewandowski, Aleksander Myszka, Małgorzata Migut, Ewelina Czenczek-Lewandowska, Robert Brodowski

**Affiliations:** 1 Medical College, University of Rzeszow, Rzeszow, Poland; 2 Departament of Maxillofacial Surgery, Fryderyk Chopin Clinical State Hospital, Rzeszow, Poland

**Keywords:** Antiplatelet therapy, Antiplatelet drugs, Tooth extraction, Topical haemostatic agents, TachoSil, Single antiplatelet therapy (SAPT), Dual antiplatelet therapy (DAPT)

## Abstract

**Background:**

Patients using antiplatelet drugs following infarctions, acute coronary syndrome or stroke pose a significant clinical problem if it is necessary to perform surgery, including dental surgery, since they are at risk of prolonged or secondary post-extraction bleeding. Discontinuation of this therapy is associated with a high risk of serious thromboembolic complications. The purpose of this study was to assess the effectiveness of TachoSil fibrin-collagen patches in stopping and preventing of secondary post-extraction bleeding in patients undergoing chronic antiplatelet therapy.

**Methods:**

The study was conducted through retrospective examination of the medical records of 153 patients using chronic antiplatelet therapy and those qualified for tooth extraction. The largest group comprised 74 patients using aspirin and clopidogrel as dual platelet antiaggregation therapy; in this group 75 tooth extractions were carried out. In all of the patients TachoSil fibrin-collagen patches and stiches were applied to the wounds resulting from tooth removal.

**Results:**

Following tooth extraction, primary bleeding was stopped in all the patients and their wounds closed via coagulation within 20–30 min. In eight cases, accounting for 4.9% of the patients, secondary bleeding occurred and was successfully stopped only by applying a pressure dressing soaked in tranexamic acid. Secondary bleeding occurred in three patients on the second day and in five patients on the third day following tooth removal.

**Conclusion:**

Topical application of TachoSil patches following tooth removal in patients using single or dual antiplatelet therapy effectively stopped bleeding and prevented secondary bleeding after tooth extraction.

## Background

Increased incidence of cardiovascular disorders and heart disease has resulted in the fact that more and more patients have to take antithrombotic drugs. Often continued further in life, the therapy is based on oral anticoagulants from the coumarin group (acenocoumarol, warfarin), new generation of oral thrombin as well as factor Xa inhibitors and antiplatelet agents [1–6]. Antiaggregants most commonly applied in both primary and secondary prevention of cardiovascular diseases include acetylsalicylic acid (ASA), clopidogrel, prasugrel, and ticagrelor [7, 8]. Acetylsalicylic acid (aspirin) inactivates cyclooxygenase, which inhibits physiological aggregation of blood platelets and development of blood clots by impairing extrinsic pathways of coagulation and clotting for the life spam of the platelet, ranging from 7 to 10 days [5, 9–11]. Antiplatelet drugs may be used in the course of single antiplatelet therapy (SAPT), or dual antiplatelet therapy (DAPT). In recent years there has been a significant increase in the number of patients requiring DAPT, i.e. aspirin (acetylsalicylic acid) as well as oral antagonists of the antiplatelet P2Y_12_ receptor for adenosine 5′-diphosphate. Data related to the year 2017, published by the European Society of Cardiology, show that annually about 1–2 million patients require DAPT due to myocardial infarction, acute coronary syndrome (ACS), following endovascular therapy on coronary vessels, i.e. angioplasty as well as angioplasty with stent implantation. Implantation of Bare Metal Stents (BMS) or Drug Eluting Stents (DES) releasing the drug for a specified duration of time requires uninterrupted antithrombotic therapy, particularly DAPT, to be continued for a period from one to 12 months. Discontinuation of antiplatelet therapy earlier than one year after stent implantation poses a risk of stent thrombosis, and the risk of death in such cases amounts to approximately 40% [5, 12–16].

Patients using both SAPT and DAPT pose a significant clinical problem if it is necessary to perform surgery, including a dental surgery. It is assumed that approximately 5% of patients starting DAPT, during the first year or later, will require an operative intervention other than cardiac surgery. This also applies to dental surgery and dental patients. Surgical procedures, involving disruption of body tissues and integument, are associated with the need to stop bleeding and achieve haemostatic balance, which is difficult in the case of patients using DAPT due to defective clotting [17–20]. Despite the guidelines of the American AHA (2017) and the Polish Cardiac Society and the Polish Dental Society for perioperative care provided to patients using anticoagulant treatment, the related issues still constitute a significant challenge in the daily practice of dental medicine and cardiology.

Of particular importance is the qualification for dental surgeries if the patient reports earlier than 12 months after the strict therapy was started. Some medical practitioners (dentists, cardiologists, family doctors), in fear of prolonged post-extraction bleeding and haemorrhagic complications, recommend that medication be discontinued, which may lead to serious thromboembolic complications [6, 7]. The choice between a risk of clots and thromboembolic complications caused by DAPT interruption and a risk of haemorrhagic complications resulting from continued antiplatelet therapy is always difficult. Most commonly it is necessary to make the decision tailored to the specific case, and to make sure the process will be the least threatening to the patient’s well-being and life [15, 21].

It is always necessary to consider whether or not it is possible to delay or postpone an intervention so that it is not performed earlier than 6–12 months after acute coronary syndrome, myocardial infarction or implantation of drug eluting stent [2, 22]. However, dental procedures often cannot be delayed because of the painfulness of the condition, acute purulent inflammation, or such random incidents as injuries, or traumatic dental injuries where it is necessary to remove the effected teeth. In such cases the procedures are performed without interruption to antithrombotic therapy, even more so because there are no standardised methods assessing platelet function and its return to the normal state in patients taking antiplatelet drugs. It is not possible to apply bridging anticoagulation in patients using antiplatelet therapy [6].

The research project was designed to investigate whether a tooth extraction procedure in which TachoSil fibrin-collagen patches are applied to the wound can safely be performed, without a need to discontinue antiaggregation therapy, and leading to normal local haemostasis, in patients requiring DAPT due to myocardial infarction, acute coronary syndrome or other cardiovascular diseases.

## Methods

### Study design

Retrospective analysis took into account medical records of 153 patients with myocardial infarction and acute coronary syndromes receiving treatment at Maxillofacial Surgery Centre at F. Chopin Regional Clinical Hospital in Rzeszow or in a private medical facility, and subjected to procedures of tooth extraction and sanitation of the oral cavity and teeth. All the 153 patients were found with active or latent pocket infections of dental nature, i.e. teeth affected by pulpitis or chronic periapical periodontitis which had to be removed. All the patients that have qualified for tooth extraction had been taking medication: ASA and/or clopidogrel (SADP or DAPT) for the duration ranging from a few months to one year from the start of cardiovascular therapy.

### Participants

The study group comprised of 82 (53.5%) males and 71 (46.5%) females, ranging in age from 52 and 77 years. The largest group, i.e. 74 patients, accounting for 48.4% of the group, used DAPT, 41 (26.6%) patients used clopidogrel monotherapy and 38 (24.8%) subjects took aspirin, as primary prevention. Table 1 presents the demographic and clinical characteristics of the patients.

**Table 1:** Characteristics of the study group

Type of medication		No. of patients	Gender		Age (years)			Indications for antiplatelet therapy					
			F	M	The youngest	The oldest	Mean	Primary prevention	Infarction	ACS	Stent implantation	Bypass	PCI
ASA	n	38	16	22	49.0	65.0	57.4	32	0	0	0	0	0
	%	24.8	22.5	26.8	–	–	–	100	0	0	0	0	0
Clopidogrel	n	41	26	15	55.0	67.0	59.8	0	14	11	10	9	3
	%	26.8	36.6	18.3	–	–	–	0	37.9	40.7	37.0	60.0	16.7
DAPT*	n	74	29	45	59.0	66.0	62.6	0	23	16	17	6	15
	%	48.4	40.8	54.9	–	–	–	0	62.1	59.3	63.0	40.0	83.3
Total	n	153	71	82	49.0	67.0	60.2	32	37	27	27	15	18
	%	100	100	100	–	–	–	100	100	100	100	100	100

^*^DAPT (Clopidogrel + ASA)

The indications for antiplatelet therapy included: coronary heart disease, ACS, as well as conditions following myocardial infarction, percutaneous coronary interventions (PCIs), implantation of stents and bypasses. Some patients used antiplatelet therapy for more than one reason, due to which the number of indications in Table 1 does not equal the number of patients.

### Setting

All the interventions were carried out as scheduled, during morning hours, and without interruption of or modifications to the antiplatelet therapy. The interventions were performed in a typical way, under local anaesthesia induced by 2% lignocaine hydrochloride, administered with a vasoconstrictor agent, and involved surgical removal of one or more teeth (to a maximum of three) in course of an intra-alveolar procedure or by formation of mucoperiosteal flap. After the tooth was extracted, curettage was applied to debride the dental socket and remove granulation and periapical tissues affected by the inflammation. A TachoSil fibrin-collagen patch was applied to the tooth socket to stop the primary bleeding and to promote formation of a stable blood clot. By achieving stable local haemostasis it was possible to prevent prolonged or secondary bleeding. Furthermore, surgical sutures were applied to the post-extraction wounds in addition to pressure dressing soaked in tranexamic acid, to be kept on for about one hour after the procedure. Following the procedure, the patients were instructed to reduce physical activity, to refrain from eating hot and hard food products, and to avoid excessive rinsing of the oral cavity so that the blood clot formed in the socket would not get damaged, flushed or torn. The patients were also advised that blood clots provide the most effective protection against secondary bleeding from post-extraction wounds. Check-ups related to secondary post-extraction bleeding as well as wound healing were recommended to be carried out on the third and fifth day following the procedure.

### Statistical methods

The clinical data collected were subjected to statistical analysis. These were computed using Statistica 13.1 software developed by StatSoft. Two-way test of significance was applied.

## Results

Analysis of the present data shows that a total of 215 teeth were removed in 153 patients using antiplatelet therapy, which produced a result of 1.405 teeth extracted per patient, i.e. approx. one and a half teeth per individual. One, two or three teeth, adjoining or located in a single maxillary or mandibular quadrant were removed in one patient during a single procedure. This resulted in a total of 163 post-extraction wounds, including 118 (72.4%) cases where one tooth was removed, 38 (23.3%) cases where two teeth were extracted as well as 7 (4.3%) cases where three teeth were extracted during a single procedure.

In the group of the patients taking aspirin as a primary prevention treatment, 73 teeth (nearly two teeth per one person—192.1%) were extracted, which resulted in 43 (58.9) wounds. During a single procedure one or two teeth were removed in a patient (19–44.2% and 18–41.9%, respectively).

In the group of patients treated with clopidogrel a total of 67 teeth were removed (on average over 1.5 teeth per person—163.4%), resulting in a total of 46 (68.7%) wounds. Most commonly one tooth or two teeth were extracted (26–56.5% and 19–41.3%, respectively). In the group of the patients requiring secondary prevention treatment with two antiplatelet drugs (DAPT), a total of 75 teeth were removed (a mean of one tooth per person—101.4%), which resulted in 74 (98.7%) wounds. In this group most commonly one tooth was removed (73–98.6%).

The percentage rate of wounds relative to the number of extracted teeth in the group of the patients taking aspirin amounted to 58.9%, in the group of patients treated with clopidogrel—68.7% and in the DAPT group—98.7%. Percentage rate of wounds relative to the number of extracted teeth differed significantly in the groups of patients treated with aspirin and DAPT (*p* < 0.001) as well as clopidogrel and DAPT (*p* < 0.001). There was no difference related to this factor between the groups treated with aspirin and clopidogrel (*p* = 0.336). The data are shown in Table 2.

**Table 2:** The number of procedures performed and teeth removed

Type of therapy	Number of patients	Number of teeth removed	Post-extraction wounds			
			Number of wounds	Number of teeth extracted		
				One	Two	Three
Aspirin	38	73	43	19	18	6
	24.8%	192.1%	58.9%	44.2%	41.9%	14.0%
Clopidogrel	41	67	46	26	19	1
	26.8%	163.4%	68.7%	56.5%	41.3%	2.2%
DAPT	74	75	74	73	1	0
	48.4%	101.4%	98.7%	98.6%	1.4%	0%
Total	153	215	163	118	38	7
	100%	140.5%	75.8%	72.4%	23.3%	4.3%

In all the cases normal haemostasis was achieved after TachoSil was applied to the post-extraction wounds, i.e. bleeding was stopped and a blood clot was developed. In all the patients (100%) bleeding was stopped within 20–30 min following the procedure. Secondary bleeding after the procedure was observed in a total of eight cases, which accounted for 4.9% of all the post-extraction wounds treated. Bleeding occurred in three patients on the second day and in five patients on the third day following tooth removal. It was mildly oozing from under the blood clot, and no additional treatment was required; the bleeding was stopped by pressing the wound with a gauze dressing soaked in tranexamic acid. In six cases (i.e. 75%) the bleeding occurred in the wounds which formed after one tooth was removed, and in 25% of cases is was observed in wounds resulting from the extraction of two teeth. No cases of bleeding were observed following removal of three teeth during a single procedure. The data related to incidence of bleeding are shown in Table 3.

**Table 3:** Incidence of post-extraction bleeding

Therapy	Number of wounds treated after tooth extraction	Post-extraction bleeding				
		Bleeding from post-extraction wounds		Bleeding from the wounds following removal of teeth		
		Present	Absent	One tooth	Two teeth	Three teeth
Aspirin	43	3	40	3	0	0
	58.9%*	7%	93%	100%	0%	0%
Clopidogrel	46	2	44	1	1	0
	68.7%	4.3%	95.7%	50%	50%	0%
DAPT	74	3	71	2	1	0
	98.7%	4.1%	95.9%	66.7%	33.3%	0%
Total	163	8	155	6	2	0
	75.8%	4.9%	95.1%	75%	25%	0%

^*^Percentage relative to the number of teeth removed

Information presented in Table 3 shows that bleeding from post-extraction wounds in the specific groups was observed at the rates of 7.0% in the group of patients taking aspirin, 4.3% in the group of patients treated with clopidogrel and 4.1% in the group of patients receiving DAPT. No statistically significant differences were identified in the incidence of bleeding between the specific groups: patients treated with aspirin versus those treated with clopidogrel, (*p* = 0.900), patients treated with aspirin versus those receiving DAPT (*p* = 0.877) as well as patients treated with clopidogrel versus those receiving DAPT (*p* = 0.991).

## Discussion

Antiplatelet drugs are most commonly applied as part of chronic primary or secondary prevention of complications associated with atherosclerosis of coronary or peripheral arteries. The use of aspirin, clopidogrel and other similar drugs is linked with a risk of prolonged bleeding following dental surgeries [9, 23]. The current study is based on a retrospective analysis focusing on 153 patients, some subjected to dual antiplatelet therapy, who had to have teeth extracted due to existing pocket infections of dental nature. The related problems are not only encountered by dental practitioners but also by family doctors or cardiology specialists consulted in regard to patients’ eligibility for surgery [24]. In the case of patients on chronic antiplatelet therapy who are eligible for tooth extraction, medical practitioners always face a dilemma whether or not to discontinue antiplatelet therapy [23]. As emphasised by numerous researchers, discontinuation of antiplatelet therapy may lead to serious consequences such as thromboembolism affecting coronary or cerebral arteries, which according to statistics in 25% of cases end with death, while 40% of such episodes may lead to permanent disability. Papanicolaou et al. described a serious case of ST-Elevation Myocardial Infarction (STEMI) associated with cardiogenic shock following discontinuation of DAPT prior to tooth removal, and with heparin bridging which proved to be ineffective and increased a risk of perioperative thrombosis [23]. It has been pointed out in the related literature that in the cases where antiplatelet therapy is not discontinued, dental surgery or tooth extraction may lead to prolonged postoperative bleeding which is difficult to stop [21, 25]. As mentioned in the introduction, a medical professional must make decisions based on his/her knowledge and clinical experience, tailored to the needs of a specific patient. However, it is always necessary to assess the risk of systemic thromboembolic complications due to discontinued therapy, and the likelihood of bleeding which can be stopped using available local haemostatic agents [26, 27].

In the current study it was hypothesised that local dressing can safely be applied to wounds resulting from tooth extraction, with no need to discontinue antiplatelet therapy, and the treatment may reduce prolonged and secondary bleeding following extraction [22, 28, 29]. Analysis of the author’s own materials show that in the group of 153 patients subjected to the procedure, secondary bleeding following the procedure was observed in eight patients accounting for 4.9% of the total group. In patients using dual therapy bleeding occurred on the second day, at a rate of 4.1%, and in the group of patients using primary prevention there were slightly more cases of bleeding, accounting for 7% of the subjects. Statistical analysis did not identify significant differences in the incidence of post-extraction bleeding relative to the primary or secondary therapy applied [19]. A review of Polish literature showed there are no articles focusing on tooth extraction and dental surgeries performed in patients using dual antiplatelet therapy. Because of its retrospective nature, the current study does not compare cases of continued and discontinued antiplatelet therapy prior to the procedure, i.e. there is no control group. The small number and low rate of cases of prolonged bleeding show that the procedure may be safely performed with no need to discontinue the medication. This outcome is associated with the fact that adequate local treatment was applied. Similar conclusions were reported by authors of experimental control studies [30, 31].

In many studies and illustrative articles, the authors emphasise the important role of local treatments applied to post-extraction wounds; the options available include a variety of local haemostatic agents, e.g. gelatine sponges, collagen sponges, oxidised cellulose, tissue adhesives, and a variety of splints and stoppers made of acrylate mass [17]. The options applied for years applied in Maxillofacial Surgery Centre at the Clinical Hospital of the University of Rzeszow include local haemostatic agents, i.e. Tissucol and Beriplast tissue adhesives; cellulose-based agents, as well as freeze-dried fibrin and collagen sealants TachoComb and TachoSil [17, 22].

The observations presented here are consistent with the findings reported by Bajkin et al. who performed tooth extractions without a risk of bleeding and without changing the algorithm of antiplatelet therapy (no interruption of the therapy); they only applied local haemostatic agents [32]. Owattanapanich et al. presented data related to efficacy and effectiveness of tranexamic acid in treatment and prevention of post-extraction bleeding in patients using anticoagulant drugs [33]. Napenas et al., based on a review of 15 studies meeting eligibility criteria, assessed the risk of bleeding after dental surgeries performed in patients subject to anti-aggregation therapy and they did not identify a higher risk of clinical complications following tooth extraction in patients using single or dual antiplatelet therapy [21]. A study by Patel et al. demonstrated that dental procedures performed in patients taking antiplatelet drugs are linked with a low risk of post-extraction bleeding; therefore, there is no need to discontinue the antiplatelet therapy [16].

The current observations support opinions presented by many researchers claiming that it is not necessary to discontinue antiplatelet therapy in connection with tooth extraction, providing that adequate and safe topical dressing is applied to the post-extraction wound. As described in the current study, TachoSil fibrin-collagen patches in our opinion effectively stopped bleeding. Likewise, a study by Lu et al. taking into account a large group of 1271 patients subject to antiplatelet therapy, taking either ASA or clopidogrel (SAPT) or both agents (DAPT), demonstrated that there is no need to discontinue antiplatelet therapy prior to scheduled tooth extraction [34].

The presented material, intended mainly for general practitioners: dentists, family doctors, cardiologists, proves that tooth extraction using the TachoSil fibrin-collagen dressing for wounds after tooth extraction in patients after myocardial infarction, acute coronary syndromes and other cardiological diseases requiring the use of DAPT antiplatelet treatment, in accordance with the recommendations of the American and European Cardiac Societies, can be safely performed without discontinuing anti-aggregation treatment and achieving normal local haemostasis.

## Limitations

Many years of observations of the authors show that surgical-dental procedures, including tooth extraction, in patients who are undergoing long-term antiplatelet treatment should be performed by a team of experienced doctors with substantive preparation and knowledge of the treatment of coagulation disorders in patients qualified for surgery in the field of dental surgery. The authors believe that knowledge of the guidelines does not limit individual decisions made in relation to a given patient, after a thorough history and physical examination. Future studies should include the study of patients who did not receive platelet anti-aggregation treatment, which will allow for comparison of both study groups and allow for a broader understanding of the topic.

## Conclusions

1. Secondary bleeding was observed in a total of eight patients, accounting for a small rate of the related complications.
2. Topical application of TachoSil, patches following tooth extraction in patients using single or dual antiplatelet therapy, effectively stops bleeding and prevents secondary post-extraction bleeding.

## Data Availability

The datasets used and/or analysed during the current study are available from the corresponding author on reasonable request.

## Abbreviations

ACS: Acute coronary syndrome
ASA: Acetylsalicylic acid
BMS: Bare Metal Stents
DAPT: Dual antiplatelet therapy
DES: Drug Eluting Stents
SAPT: Single antiplatelet therapy
STEMI: ST-Elevation Myocardial Infarction
PCIs: Percutaneous coronary interventions
